# A data-driven approach for the discovery of biomarkers associated with thyroid eye disease

**DOI:** 10.1186/s12886-021-01903-9

**Published:** 2021-04-09

**Authors:** Huihui Zou, Weiwei Xu, Ying Wang, Zhihong Wang

**Affiliations:** 1 Department of Ophthalmology, Dezhou People’s Hospital, Dezhou, 253000 China; 2 Department of Ophthalmology, Dezhou Women’s and Children’s Hospital, Shandong, China

**Keywords:** Thyroid eye disease, Autoimmune disease, Biomarker, Graves’ disease

## Abstract

**Background:**

Thyroid eye disease (TED) is the most common autoimmune disease and usually occurs in patients with hyperthyroidism. In this disease, eye-related tissue, such as eye muscles, eyelids, tear glands, etc., become inflated, which causes the eyes and eyelids to become red, swollen, and uncomfortable. The pathophysiology of this disease is still poorly known.

**Aim:**

This study aims to discover potential biomarkers and regulatory pathways of TED which will not only help to diagnose the disease and understand orbital involvement in thyroid dysfunction but also provide an insight for better therapeutics.

**Methods:**

We applied a data-driven approach by combining gene biomarkers both from published literature and computationally predicted from microarray gene expression data. Further, the DAVID tool is used for Gene Ontology-based enrichment analysis.

**Results:**

We obtained a total of 22 gene biomarkers, including 18 semi-automatically curated from the literature and 4 predicted using data-driven approaches, involved in the pathogenesis of TED that can be used as potential information for therapeutic targets. Further, we constructed a regulatory pathway of TED biomarkers comprises of 310 connected components, and 1134 interactions using four prominent interaction databases.

**Conclusion:**

This constructed pathway can be further utilized for disease dynamics and simulation studies.

**Supplementary Information:**

The online version contains supplementary material available at 10.1186/s12886-021-01903-9.

## Introduction

Thyroid eye disease (TED), also known as Graves’ eye disease (GED), or Graves’ ophthalmopathy (GO), is an autoimmune condition in which immune cells attack the thyroid gland due to secretion of excess amount of thyroid-stimulating hormone (TSH). These excess hormones increase metabolism (hypermetabolism) which is characterized by fast heartbeat, palpitation, profuse sweating, high blood pressure, heat intolerance, weight loss, etc. [1, 2] The hyperthyroidism leads to the eye protruded from eye orbit due to inflammatory disorder and also leads to permanent facial disfigurement. It causes swelling of muscle, fat, tissues, i.e. periorbital tissues leading to proptosis [3]. In fact, autoimmune attack generally targets the eye muscles because these tissues contain proteins that seem similar to the immune system as those of the thyroid gland [4, 5]. According to the ReportLinker report, the global treatment market of TED is expected to pump up from USD 211.49 Million in 2019 to USD 344.19 Million by the end of 2025 [6]. In another report published by MarketWatch News, the TED market projected a compound annual growth rate (CACG) of 8.53% during the forecast period of 2020–2026 [7].

In TED, tissues around the eye are attacked, leading to inflammation and swelling, which causes redness and pain, puffiness around the eyes, erythema, conjunctivitis, proptosis, and upper eyelid retraction [8]. Environmental factors like smoking and radioactive iodine are more predisposed to TED [9]. There are many other factors but smoking has a major influence [10]. Smoking induces thyroid functional changes, like a decrease or increase in thyroid hormones and also the risk of thyroid cancer [11]. Like thyroid disease, women are more vulnerable to TED than males with the female to male ratio of 4:1 [12]. Immune cells attack the periorbital tissues that lead to the expansion of eye muscles or fat [2, 12]. Hyperthyroidism leads to an overactive thyroid gland, i.e., more secretion of TSH. The TSH helps to maintain body metabolism, and it’s over secretion develops several consequences like high blood pressure, fatigue, weight loss, and irritabilitye [13]. Further, autoimmune cells attack the enlarged thyroid gland as well as eye muscles and periorbital tissue. These tissues contain proteins that appear similar to the thyroid gland, like thyroid-stimulating hormone receptor (TSH-R), target as immune assault [14]. The progressive eye swelling may trigger acute pressure inside the eye socket, pressure-pain which worsens movements of the eye, decreased vision when swollen tissues push on the optic nerve [2].

The TED is diagnosed by a blood test, thyroid computed tomography (CT) scan, magnetic resonance imaging (MRI) scan, radioactive iodine uptake test, low thyroid TSH test, positive thyroid-stimulating immunoglobulin (TSI) test, increase free thyroxine (T4), and elevated anti-peroxidase [15, 16]. Since TED occurs due to the immune system’s attack on the healthy tissues, therefore, treatment of the thyroid gland does not improve eye disease [17]. Some of the prevalent methods of treatment of Graves’ disease are anti-thyroid drugs, such as *Thionmaids*, *Methimazole*, *Teprotumumab* (under clinical trial and study by FDA) [18] or beta-blockers (such as *Propranolol*, *Atenolol*, *Metoprolol*), use of radioactive iodine, or surgery [19]. These treatments are based on age and the degree of illness of the patient [20]. The use of anti-thyroid drugs, which reduces the release of thyroids hormones, is the least invasive method to treat Graves’ disease. Radioactive iodine therapy is the most common method to treat Graves’ disease in the United States. The definite treatment of Graves’ disease is thyroidectomy, a surgery to remove the thyroid gland [21, 22]. As far as TED is concerned, mild cases may be treated with sunglasses, artificial tears, or ointments. However, more serious cases may be treated with corticosteroids which reduce swelling of tissues around the eyes. Orbital therapy and orbital decomposition surgery are also used to treat TED [23, 24].

The molecular mechanism underpinning TED is gradually becoming clearer due to advancements in both experimental and computational techniques. The availability of large-scale biological data (i.e., multi-omics) offers a paradigm shift from sub-optimal treatment to optimal targeted therapy [25]. Biomarkers are of pivotal importance which serves as a useful noninvasive tool in the clinical armamentarium for disease studies including its diagnosis, prevention, drug target identification, designing drug for a particular receptor, and biological processes to a therapeutic intervention [26]. It can be genes, mRNAs, and metabolites. In the case of TED, a set of biomarkers were identified in immunogenetics, hormones, antibodies, cytokines, urine, orbital fat, and tear. Peroxisome proliferation activation receptor gamma (PPAR-γ) activation leads to an eye’s protrusion. Triiodothyronine (T3) and Thyroxine (T4) dysfunction secretion and antibodies (TRAbs) levels are associated with the severity of TED [9, 27]. IL-1^β^, IL-10, IL-8, C-C chemokine ligand 20 (CCL20), IL-17 are the pro-inflammatory cytokines. Polymorphism of IL-10 is associated with the incidence of TED [28]. Soluble vascular cell adhesion molecules-1 (sVCAM-1) and intercellular adhesion molecules-1 (ICAM-1) are elevated in the blood of TED patients [29]. CLAT-4 immunoglobulin functions as an immune checkpoint and downregulates immune responses [30]. Further, CD152, an expression product of CTLA-4 genes, downregulates T-cell activation [31]. IL-1^β^ and IL-6 level in orbital fat associated with the smoking status of TED patients [32]. IFN-γ is differentially upregulated in TED, and platelet-derived growth factor (PDGF) responsible for the physiological event in TED [32]. PDGF and IL-1^β^ induce adipogenesis through the enzyme cyclooxygenase-2 (COX-2). Levels of TSHR-Ab are a useful tool for the measurement of TED [33]. PAI-1 and SERPINE1 regulate the proteolytic activity [34]. Wong and colleagues [35] reported the association of IL-1A and other IL genes with Graves’s Ophthalmopathy (GO). Tear proteins, like S100A4 and PIP, also serve as biomarkers to predict ocular and systemic disease progression [36]. PTPN22 (protein tyrosine phosphatase) shows negative regulation of T-cell activation [37]. NF-kB (nuclear factor kappa-B) is a transcriptional factor activated by various cytokines. Other genetic factors like TLR-9, CD86, CD103, glucocorticoid receptors, CTLA-4, TNF-alpha, HLA-DRB-1 are also associated with TED [38]. A review on biomarkers associated with TED can be found in Turck et al. [32] and Longo et al. [34]

Even though few studies have been carried out to identify gene biomarkers in TED, but its study at the genome-wide scale is lacking. Further, the possible regulatory pathway of biomarkers in TED has not been studied earlier. Hence, in the present study, we applied a data-driven approach to detect gene biomarkers in TED, which combines biomarkers both from reported literature as well as we computationally predicted using microarray gene expression profiles. Further, a regulatory pathway of biomarkers is constructed followed by various Gene Ontology-based enrichment analyses. This regulatory pathway can be further utilized for disease dynamics, molecular docking, and simulation studies.

## Methods

**Fig. 1:**
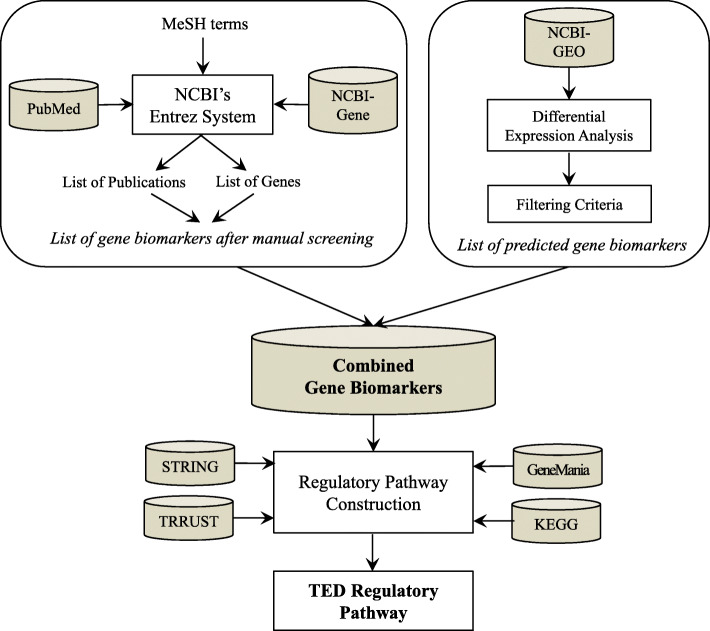
Methodological pipeline

The thyroid eye disease (TED) is a complex disease having an overlap with other Grave’s disease and there are several molecular players involved. Here, we constructed a systematic network of gene regulatory pathways of TED. The network is based in semi-automated curate literature-based information reported to be gene biomarkers of TED and predicted information using high-throughput gene expression data. The constructed network has been topological, gene ontology (GO) and tissue-specifically analyzed to translate it into meaningful disease-specific and tissue-specific markers and can be utilized as targets in the diagnosis and therapy of TED. The methodological pipeline adopted in this study is depicted in Fig. 1.

### Extraction of experimentally determined biomarkers

To extract TED genes from published literature, we used the Entrez System of the National Center for Biotechnology Information (NCBI) and performed a query with the MeSH (Medical Subject Headings) terms “**((thyroid eye disease) AND biomarker)**”. The Entrez (https://www.ncbi.nlm.nih.gov/) is an online cross-database search system that helps the users to perform a global query in NCBI’s various genomics, genes, proteins, genetics, literature, and other health sciences databases. The search results provide a summary of hits in various NCBI databases. We recorded the list of genes from the NCBI-Gene database and a list of published literature in NCBI-PubMed for manual screening of biomarker genes in TED. The manually screened biomarker genes were cross-checked by two independent reviewers.

### Prediction of gene biomarkers using gene expression profiles

To predict gene biomarkers of TED, we took microarray gene expression data from the NCBI-GEO database (https://www.ncbi.nlm.nih.gov/geo/query/acc.cgi?acc=GSE58331). In this dataset, RNA was extracted and analyzed with Affymetrix from anterior orbit and lacrimal gland tissues of the collected biopsies. The data contains gene expression of various inflammatory diseases including TED, but we considered only TED gene expression data from anterior orbit tissue for this study. The considered datasets contain 27 TED samples and 22 control samples, both from anterior tissues of men as well as women.

### Construction of gene regulatory pathway

Molecular interaction data of the identified gene biomarkers were collected from four major databases including STRING [39], GeneMania [40], TRRUST [41], and KEGG Pathway [42]. These interaction data were merged to construct a consolidated gene regulatory pathway for further downstream analysis.

### GO enrichment analysis

Gene Ontology (GO) enrichment is performed to study various enrichment analyses of a given gene set, i.e., a list of gene biomarkers, that finds which GO terms are either over-represented or under-represented using stored annotations in the database. We utilized DAVID 6.8 tool [43] for studying GO enrichment analysis, functional category analysis, disease enrichment, and disease class enrichment analysis, and KEGG pathway enrichment analysis. The DAVID 6.8 server can be accessed from https://david.ncifcrf.gov/.

## Results

After performing the query with the MeSH term “**((thyroid eye disease) AND biomarker)**” in NCBI’s Entrez System, we obtained a list of 24 genes in NCBI’s Gene database along with a list of 285 publications in PubMed. We manually screened these 24 genes to verify their role as biomarkers in TED. Further, we also manually screened all the obtained publications to obtain more reported biomarkers in TED. Finally, after the manual screening of each gene and reported literature, we shortlisted a list of 18 experimentally verified genes reported to be involved in the pathogenesis of TED, as shown in Table 1 along with PubMed ID (PMID) of supporting literature, and its involvement in various pathways. To predict gene biomarkers using microarray gene expression data of TED (GEO Accession No. GSE58331), we executed the GEO2R tool on both TED samples and normal samples. In other words, we performed a case-control analysis to find out differentially expressed genes (DEGs) that can be utilized as disease biomarkers. We applied the fold-change statistics to compute DEGs, along with several other statistics including *p*-value, and false discovery rate (FDR). To filter significant genes that may be the potential gene biomarkers of the disease under study, we applied a threshold of p-value <=0.05 and have at least two-fold change (i.e., − 1.0 > = *log*FC > = + 1.0) in their gene expression, as generally applied and suggested by researchers [44–47]. Further, duplicate genes and genes with missing names and statistical values were removed from the list. In this way, we obtained a list of 63 genes for down-stream analysis. To further narrow down and perform significant analysis of predicted gene biomarkers, we shortlisted only those genes which are transcription factors (TFs), and associated with autoimmune or thyroid disease, and/or involved in autoimmune or thyroid disease KEGG pathway, and/or GO enriched with the term “thyroid” or “autoimmune” as a biological process, shown in Table 2. For this purpose, we utilized the TRRUST v2 database [41]. Hence, we obtained a total of 22 gene biomarkers, including 18 semi-automatically curated from the literature and 4 predicted, involved in the pathogenesis of TED that can be used as potential information for therapeutic targets.

**Table 1:** List of experimentally verified and reported gene biomarkers of TED

S.No.	GeneID	Gene Symbol	Gene Description	KEGG Pathway / Disease Term (Selected)	Map Location	Genomic Nucleotide Accession	Exon count	PMID
1	3091	HIF1A	hypoxia inducible factor 1 subunit alpha	Thyroid hormone signaling pathway (hsa04919), Thyroid cancer (hsa05216)	14q23.2	NC_000014.9	16	27,610,652
2	3576	CXCL8	C-X-C motif chemokine ligand 8	Cytokine-cytokine receptor interaction (hsa04060), Chemokine signaling pathway (hsa04062), NF-kappa B signaling pathway (hsa04064)	4q13.3	NC_000004.12	4	31,149,053, 31,059,842
3	3627	CXCL10	C-X-C motif chemokine ligand 10	Cytokine-cytokine receptor interaction (hsa04060), Chemokine signaling pathway (hsa04062), TNF signaling pathway (hsa04668)	4q21.1	NC_000004.12	4	31,059,842, 24,999,581, 22,378,921
4	3586	IL10	interleukin 10	Autoimmune thyroid disease (hsa05320), Cytokine-cytokine receptor interaction (hsa04060), Intestinal immune network for IgA production (hsa04672)	1q32.1	NC_000001.11	5	30,018,377, 21,067,483, 23,754,356
5	3605	IL17A	interleukin 17A	Cytokine-cytokine receptor interaction (hsa04060), Th17 cell differentiation (hsa04659), Inflammatory bowel disease (hsa05321)	6p12.2	NC_000006.12	3	24,994,866
6	50,616	IL22	interleukin 22	Cytokine-cytokine receptor interaction (hsa04060), Jak-STAT signaling pathway (hsa04630), Th17 cell differentiation (hsa04659), Inflammatory bowel disease (hsa05321)	12q15	NC_000012.12	6	28,839,453
7	3565	IL4	interleukin 4	Tyrosine metabolism (hsa00350), Phenylalanine metabolism (hsa00360),Tryptophan metabolism (hsa00380)	5q31.1	NC_000005.10	5	21,067,483
8	3569	IL6	interleukin 6	Cytokine-cytokine receptor interaction (hsa04060), Jak-STAT signaling pathway (hsa04630), Th17 cell differentiation (hsa04659)	7p15.3	NC_000007.14	6	30,018,377
9	3596	IL13	interleukin 13	Cytokine-cytokine receptor interaction (hsa04060), Jak-STAT signaling pathway (hsa04630), Th1 and Th2 cell differentiation (hsa04658)	5q31.1	NC_000005.10	6	30,018,377
10	3479	IGF1	insulin like growth factor 1	EGFR tyrosine kinase inhibitor resistance (hsa01521), Signaling pathways of MAPK (hsa04010), Ras (hsa04014) Rap1 (hsa04015), HIF-1 (hsa04066), FoxO (hsa04068), etc.	12q23.2	NC_000012.12	7	31,313,753, 25,560,705, 29,273,685, 26,188,228
11	7040	TGFB1	transforming growth factor beta 1	MAPK signaling pathway (hsa04010), Cytokine-cytokine receptor interaction (hsa04060), FoxO signaling pathway (hsa04068), Cell cycle (hsa04110)	19q13.2	NC_000019.10	7	20,181,974
12	3458	IFNG	interferon gamma	Cytokine-cytokine receptor interaction (hsa04060), Th1 and Th2 cell differentiation (hsa04658), Th17 cell differentiation (hsa04659)	12q15	NC_000012.12	4	20,181,974, 24,999,581, 23,754,356, 26,089,587, 22,378,921
13	7057	THBS1	thrombospondin 1	Rap1 signaling pathway (hsa04015), p53 signaling pathway (hsa04115), TGF-beta signaling pathway (hsa04350)	15q14	NC_000015.10	22	31,173,926, 26,154,823
14	7253	TSHR	Thyroid stimulating harmone receptor	Autoimmune thyroid disease (hsa05320),Thyroid hormone synthesis (hsa04918), cAMP signaling pathway (hsa04024)	14q31.1	NC_000014.9	12	29,771,755, 28,127,991, 12,790,806
15	7124	TNF-Alpha	tumor necrosis factor-α	Cytokine-cytokine receptor interaction (hsa04060), T cell receptor signaling pathway (hsa04660), MAPK signaling pathway (hsa04010)	6p21.33	NC_000006.12	4	30,018,377, 26,089,587, 22,378,921
16	3107	HLA-C	major histocompatibility complex, class I, C	Autoimmune thyroid disease (hsa05320), Endocytosis (hsa04144), Phagosome (hsa04145)	6p21.33	NC_000006.12	8	17,521,325,
17	1471	CST3	cystatin C	Salivary secretion (hsa04970), Age-related macular degeneration (H00821), Cerebral amyloid angiopathy (H01185)	20p11.21	NC_000020.11	4	30,018,377, 28,702,253, 25,829,418
18	12	SERPINA3	serpin family A member 3	AACT, ACT, GIG24, GIG25	14q32.13	NC_000014.9	5	30,018,377

**Table 2:** List of predicted TF gene biomarkers in TED

S.No.	Gene names	Fold-change	Regulation type	Disease ontology term	KEGG pathway disease term	GO biological process term
1.	EGR1	3.0166	Up	autoimmune disease	Autoimmune thyroid disease (hsa05320), Thyroid hormone signaling pathway (hsa04919)	immune response
2.	FOS	3.0109	Up	autoimmune disease, thyroid gland disease	Autoimmune thyroid disease (hsa05320), Thyroid hormone signaling pathway (hsa04919)	innate immune response, immune response
3.	MAF	2.4210	Up	--	Autoimmune thyroid disease (hsa05320),	regulation of immune response, immune response
4.	NR4A1	2.0338	Up	Thyroid carcinoma, Diabetic Retinopathy, autoimmune disease	–	–

**Fig. 2:**
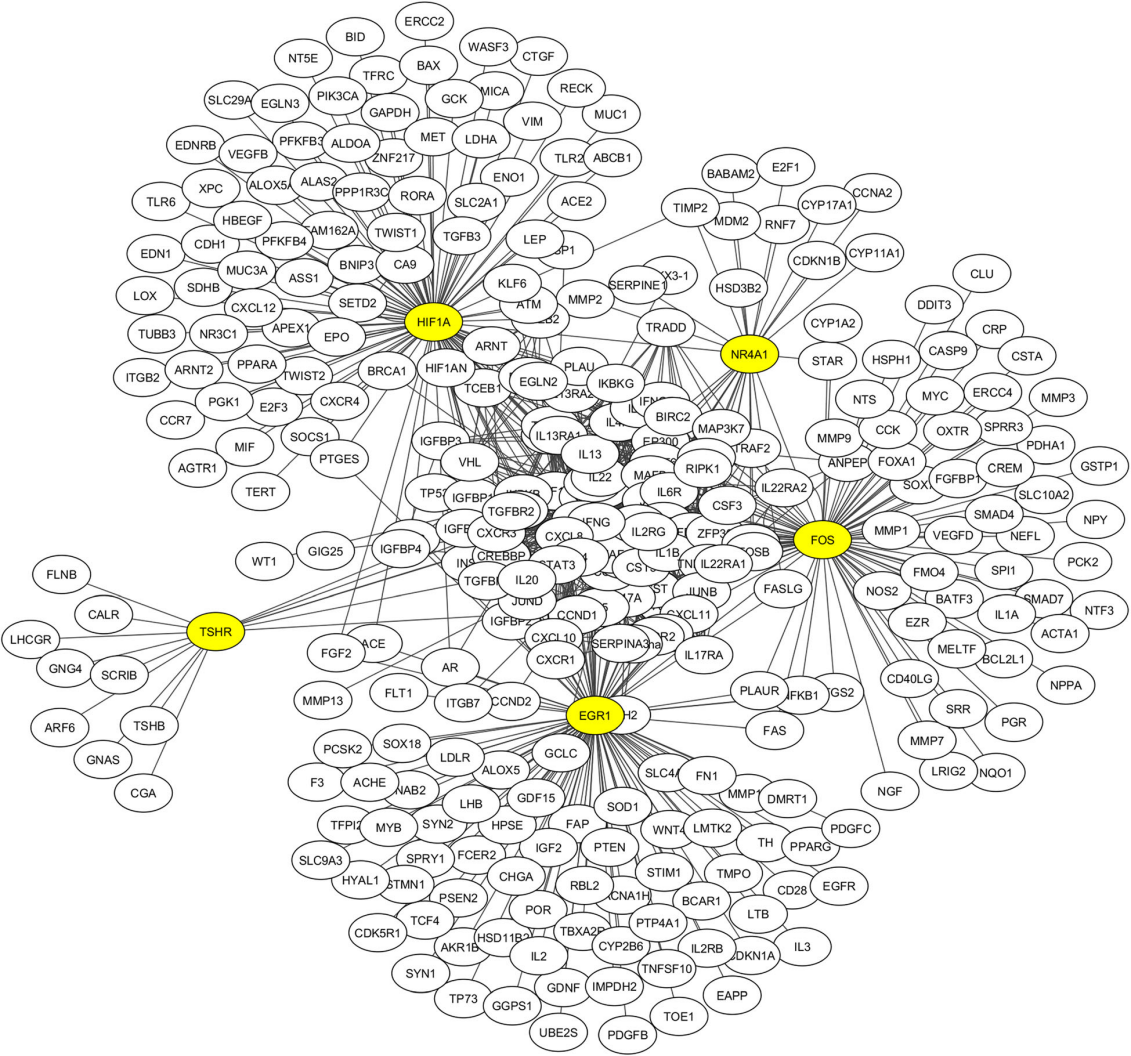
Constructed gene interaction of the biomarkers of TED, having several hubs including HIF1A, NR4A1, FOS, TSHR, etc.

To construct a consolidated gene regulatory path of TED biomarkers, we retrieved and merged the interaction of identified 22 biomarkers from four major databases. All the duplicate interactions were eliminated. The network in Fig. 2 shows the identified regulatory pathway of TED biomarkers consists of 310 connected components, 1134 interactions, the average number of neighbors of 7.3, two self-loops, and a clustering coefficient of 0.234. This network is scale-free and follows power-law. To further narrow down the list of the most significant genes for TED, we performed GO enrichment analysis, functional category analysis, disease enrichment, and disease class enrichment analysis, and KEGG pathway enrichment analysis using the DAVID tool. The terms and keywords used for this enrichment analysis and their subsequent results are presented in Table 3. To focus our analysis on TED, we considered GO enrichment terms as “inflammatory response” and “immune response”, and analysis results show that most of the identified gene biomarkers are enriched with either of these two terms or both (Table 3). Similarly, in disease enrichment class analysis, most of the disease terms are enriched with “vision” or “immune” or both (Table 3). In the KEGG pathways analysis, most of the genes are either enriched with the term “autoimmune thyroid disease” or “cytokine-cytokine receptor interaction” (Table 3). Hence, these deeper analysis results provide that the identified gene biomarkers are involved in the pathogenesis of TED.

**Table 3:** Results of various enrichment analysis of identified gene biomarkers

Genes	Go Enrichment Term		Functional Categories (UP_KEYWORDS)	Disease enrichment class (GAD_DISEASE_CLASS)		Disease enrichment (GAD_DISEASE)			KEGG Pathways (Term)	
	inflammatory response	Immune Response	Cytokine	Vision	Immune	autoimmune disease	grave’s disease	Thyroid associated ophthalmopathies	autoimmune thyroid disease	Cytokine-cytokine receptor interaction
HIF1A	풙	풙	풙	**✓**	**✓**	풙	풙	풙	풙	풙
CXCL8	**✓**	**✓**	**✓**	**✓**	**✓**	풙	**✓**	풙	풙	**✓**
CXCL10	**✓**	**✓**	**✓**	풙	**✓**	풙	풙	풙	풙	**✓**
IL10	**✓**	**✓**	**✓**	**✓**	**✓**	**✓**		**✓**	**✓**	**✓**
IL17A	**✓**	**✓**	**✓**	풙	**✓**	풙	풙	풙	풙	**✓**
IL22	**✓**	**✓**	**✓**	풙	**✓**	풙	풙	풙	풙	**✓**
IL4	풙	**✓**	**✓**	**✓**	**✓**	**✓**	**✓**	**✓**	**✓**	**✓**
IL6	**✓**	**✓**	**✓**	**✓**	**✓**	**✓**	**✓**	풙	풙	**✓**
IL13	**✓**	**✓**	**✓**	**✓**	**✓**	풙	**✓**	풙	풙	**✓**
IGF1	풙	풙	풙	**✓**	**✓**	풙	풙	풙	풙	풙
TGFB1	**✓**	풙	풙	**✓**	**✓**	**✓**	풙	**✓**	풙	**✓**
IFNG	풙	**✓**	**✓**	**✓**	**✓**	**✓**	**✓**	풙	풙	**✓**
THBS1	**✓**	**✓**	풙	풙	풙	풙	풙	풙	풙	풙
TSHR	풙	풙	풙	**✓**	**✓**	풙	**✓**	풙	**✓**	풙
TNF-Alpha	풙	풙	풙	풙	풙	풙	풙	풙	풙	풙
HLA-C	풙	**✓**	풙	**✓**	**✓**	**✓**	풙	풙	**✓**	풙
CST3	풙	풙	풙	**✓**	풙	풙	풙	풙	풙	풙
SERPINA3	**✓**	풙	풙	**✓**	**✓**	풙	풙	풙	풙	풙
EGR1	풙	풙	풙	**✓**	**✓**	풙	풙	풙	풙	풙
FOS	**✓**	풙	풙	풙	풙	풙	풙	풙	풙	풙
MAF	풙	풙	풙	풙	풙	풙	풙	풙	풙	풙
NR4A1	풙	풙	풙	풙	풙	풙	풙	풙	풙	풙
Gene count	**11**	**11**	**9**	**14**	**16**	**6**	**6**	**3**	**4**	**10**

## Discussion

The disease biomarkers is an important noninvasive tool in the clinical armamentarium for the study of diseases. This study focuses on the discovery of potential biomarkers and regulatory pathways in TED that can be utilized for disease diagnosis and helps us know insight for better therapeutics. Our analysis of combined gene biomarkers, both curated from published literature and computationally predicted using microarray gene expression data, discern its involvement in TED and other thyroid-associated ocular diseases. Our data-driven analysis identified a list of 9 Cytokines (CXCL8, CXCL10, IL10, IL17A, IL22, IL4, IL6, IL13, and IFNG) which are reported to increase the volume of orbital tissue [32]. Further, enrichment analysis identified 11 genes involved in the inflammatory response, 11 genes involved in immune response, and 10 genes are enriched in cytokine-cytokine receptor interaction (refer to Table 3). Cytokine IL10 is pro-inflammatory and its polymorphism has been reported to be associated with the incidence of TED [28]. IFNG is also reported to be differentially upregulated in TED [32]. Levels of TSHR-Ab are a useful tool for the measurement of TED [33]. Our major analysis results, in-line with the reported literature, are summarized in the following section.

### TSHR as an autoantigen in TED

TSHR is a glycoprotein hormone receptor and a member of the G protein-coupled receptor family. It has a ligand-binding extracellular domain, intracellular domain, and a transmembrane domain. The thyroid-stimulating immunoglobulins (TSI), also called thyroid-stimulating antibodies (TSAb), and TSH bind to TSHR which leads to activation of the receptor and unregulated production of thyroid hormones [48]. Besides thyroid epithelium, TSHR is found in several connective tissues and adipose depots. The mRNA expression level of TSHR is higher in orbital fibroblasts from TED patients. In TED, TSIs can activate TSHR that signals the production of IL-6 [49]. The KEGG pathway enrichment analysis discerns that TSHR is enriched in various pathways including autoimmune thyroid disease (hsa05320), Thyroid hormone synthesis (hsa04918), and cAMP signaling pathway (hsa04024). `.

### TSHR-IGF1R cross-talk

In several studies, it is reported that TSHR is the main target of stimulatory autoantibodies in the pathogenesis of TED, and stimulatory IGF1R autoantibodies cross-talk with TSH [50–52]. In fact, signaling initiated from either of these two receptors can be controlled by inhibiting the activity of IGFR1 [53]. These two make a physically and functionally interactive complex within orbital fibroblasts, and inhibition of IGF1R reduces TSH-dependent signalling [25]. Smith et al. [54] call TSHR-IGF1R cross talk as “*partners of crime*”, while Wiersinga [55] calls it “*an unfortunate marriage between TSHR and IGFR1*”. Teprotumumab is an IGF1R inhibitor that interrupts the key molecular mechanism of TED pathogenesis and is reported to have significant potential to reduce disease manifestations [25, 56, 57].

### Cytokines and chemokines in TED

Cytokines and chemokines may induce the expression level of immunomodulatory proteins in orbital fibroblasts and may contribute to disease progression [58]. Cytokines are small proteins that are important in certain diseases, especially immune response, inflammation, and host response to infection. They are involved in various cell signaling including autocrine, paracrine, and endocrine signaling, known as immune-modulating agents. Interleukins (IL), chemokines, interferons (TFNs), and tumor necrosis factors (TNFs) are known as cytokines. Chemokines are small cytokines that produce various types of cells as immune cells that include four subfamilies: CXC, CC, XC3C, and XC. In TED, orbital tissue remodeling is carried out due to cytokine-dependent fibroblast activation. The literature reports that cytokines (IL-4, IL-6, IL-10, IL-13, IL-17A, IL-22, TNFA, IFNG) and chemokines (CXCL8, CXCL10) were found in extraocular muscles and fat of TED patient [59], and differential modulation of CXCL8 versus CXCL10 by cytokines [59]. In our study, some of the identified cytokines (IL10, IL17A, IL22, IL4, IL6, IL13, and IFNG) and chemokines (CXCL8, CXCL10) gene biomarkers of TED are aligned with these findings.

## Conclusion

Thyroid eye disease (TED) is an autoimmune disease and hyperthyroidism where the tissue around the eye is attacked, leading to inflammation and swelling, which causes redness and pain, puffiness around the eyes, erythema, conjunctivitis, proptosis, and upper eyelid retraction. Among the several factors, smoking has a major influence on TED. Like hyperthyroidism, women are more vulnerable to TED than males with a female to male ratio of 4:1**.** Due to advancements in high-throughput and computational techniques, the molecular mechanism underpinning TED is gradually becoming clearer. The availability of large-scale biological data (i.e., multi-omics) offers the better discovery of biomarkers which serves as a useful noninvasive tool in the clinical armamentarium for disease studies including its diagnosis, prevention, drug target identification, designing drug for a particular receptor, and biological processes to a therapeutic intervention.

In this study, we applied a data-driven approach to detect gene biomarkers in TED, which combines biomarkers from both reported in the literature as well as we computationally predicted. Further, a regulatory pathway of biomarkers is constructed followed by various Gene Ontology-based enrichment analyses. This regulatory pathway provides an insight into the regulation mechanism in TED. Our study reports 22 gene biomarkers involved in the pathogenesis of TED that can be used as potential information for therapeutic targets. Further, we constructed a regulatory pathway of TED biomarkers consists and performed GO enrichment analysis, functional category analysis, disease enrichment, and disease class enrichment analysis, and KEGG pathway enrichment analysis using the DAVID tool. In this future work, you may perform a deeper analysis of biomarkers and constructed networks, perform molecular docking, and simulation studies against identified potential biomarkers.

## Supplementary Information

**Additional file 1.**

## Data Availability

The datasets generated and/or analysed during the current study are available in the Entrez (https://www.ncbi.nlm.nih.gov/) and NCBI-GEO database (https://www.ncbi.nlm.nih.gov/geo/query/acc.cgi?acc=GSE58331).
